# Pediatric paragonimiasis: a retrospective analysis of cases from a county in south-west China

**DOI:** 10.3389/fped.2023.1143262

**Published:** 2023-05-17

**Authors:** Yong-xin Jiang, Gong-qiang Li, Cheng-jing Pan, Zhong-qiu He, Chao Wang, Qi-ru Mu, Lu-lu Cao

**Affiliations:** ^1^ Department of Pediatrics, Yanjin Country People’s Hospital, Zhaotong, China; ^2^ Department of Pediatric Intensive Care Unit, Xinhua Hospital Affiliated to Shanghai Jiaotong University School of Medicine, Shanghai, China

**Keywords:** paragonimiasis, children, pleural effusion, peritoneal effusion, pericardial effusion

## Abstract

**Introduction:**

The clinical manifestations of paragonimiasis are diverse and non-specific, and can easily lead to misdiagnosis. We aimed to analyze the clinical manifestations, laboratory features, treatment, and clinical outcome of children with paragonimiasis in order to improve recognition of this disease and avoid misdiagnosis.

**Methods:**

Children diagnosed with paragonimiasis from August 2016 to July 2022 were included in the study. Information on population informatics, medical history, and laboratory features was extracted from case data. The clinical features of paragonimiasis were retrospectively analyzed.

**Results:**

A total of 45 children were included in this study. All children had, at least, one risk factor. The clinical features mainly included fever, cough, pleural effusion, peritoneal effusion, and subcutaneous nodules. The main imaging findings were alveolar exudation, peritoneal effusion, pleural thickening, and local nodules. The “tunnel sign” finding on computed tomography (CT)/magnetic resonance imaging (MRI) was helpful in establishing the diagnosis of paragonimiasis. After praziquantel treatment, most of the children improved, and one child with cerebral paragonimiasis experienced sequelae.

**Conclusion:**

Most children with paragonimiasis have a good prognosis, but few children can experience sequelae. Avoidance of untreated water and raw food is a simple, feasible, and effective preventive measure.

## 1. Introduction

Paragonimiasis is an important food-borne parasitic zoonosis caused by *Paragonimus spp* (1). It was estimated that there were about 23.2 million cases of paragonimiasis worldwide in 2005, including about 5 million heavily infected patients and cases that resulted in death (2, 3). In China, the national survey from 2001 to 2005 showed that the positive rate of serological tests for *Paragonimus* infection was 1.71%, and the positive rate of serological tests was higher in children under 9 years of age (4). Also, the prevalence of paragonimiasis was higher in Sichuan, Yunnan, and Chongqing due to their special dietary habits (5).

Human paragonimiasis is usually caused by drinking water containing metacercariae or consumption of raw or uncooked food contaminated with *Paragonimus spp* (1). There are more than 50 species of *Paragonimus spp*. In China, the main species are *Paragonimus westermani* and *Paragonimus* *skrjabini* (6). *Paragonimus skrjabini* and *Paragonimus* *westermani* have a similar life cycle.

**Figure 1:**
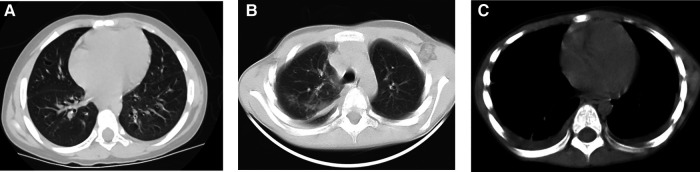
A 5-year-old boy had habit of drinking untreated water. CT of the lungs showed cystic areas (**A**), pulmonary exudation (**B**) and pleural effusion (**C**).

Paragonimiasis is still one of the diseases that affect the health of children, especially in southwest China. The nonspecificity of clinical features can easily result in this disease condition being misdiagnosed as other diseases, such as tuberculosis and cancer (2, 3), especially in a situation in which the medical personnel do not have enough knowledge about the disease or do not pay enough attention to it. This can lead to delayed treatment and even a poor prognosis, as well as an increased medical burden. Thus, the aim of this retrospective study was to improve the recognition of paragonimiasis and reduce misdiagnosis, by analyzing the clinical manifestations, laboratory features, treatment, and prognosis of children with paragonimiasis during the past six years.

## 2. Materials and methods

### 2.1. Diagnosis of paragonimiasis

The diagnostic criteria were in accordance with the “Diagnosis of Paragonimiasis” (NO. WS 380-2012), which was drafted by the National Health Commission of the People's Republic of China in 2012 (6). In brief, the diagnosis of paragonimiasis was based on the risk factor, clinical manifestations, laboratory examination, and imaging examination. Suspected cases were defined as those having clinical features, risk factors and significantly elevated peripheral blood eosinophil ratios or absolute values.

Clinically diagnosed cases were defined as suspected cases that had one of following positive test results: (1) a positive intradermal test; (2) positive serum immunology test; (3) abnormal findings in imaging examination; and (4) biopsy showing characteristic pathological changes. Cases were defined as confirmed cases if *Paragonimus* eggs were found in the sputum or stool, or if *Paragonimus* worms or eggs were found in a subcutaneous mass or other living tissue and body fluids.

#### 2.1.1. Clinical classification of paragonimiasis

The clinical classification was divided into thoracopulmonary paragonimiasis and extrapulmonary paragonimiasis. Patients with thoracopulmonary paragonimiasis may present with cough, chest pain, rusty or blood-stained sputum, pleural lesions, and other symptoms and signs. Patients with extrapulmonary paragonimiasis may have a subcutaneous mass, abdominal discomfort, and abnormal symptoms and signs of the nervous system. Some mildly infected patients may have no obvious clinical symptoms and signs.

#### 2.1.2. Risk factors

The risk factor extracted the following: (1) previous history of consumption of raw or semi-raw freshwater crabs and crayfish contaminated with *Paragonimus*, (2) history of consumption of raw or semi-raw mammalian meat (e.g., boar meat and spiny frog), and (3) history of drinking raw or untreated stream water.

### 2.2. Study population and data collection

From August 2016 to July 2022, 45 children with paragonimiasis infection were admitted to the department of pediatrics, Yanjin County People's Hospital, which is located in a mountainous county with multi-ethnic communities in southwest China. Firstly, patients diagnosed with paragonimiasis, pleural effusion, peritoneal effusion, and pericardial effusion were selected from the case system. Secondly, children with paragonimiasis whose etiological test results were positive for the antibody to *Paragonimiasis* (ELISA, Novo medical, Co. LTD) or in whom eggs/bodies were detected in the tissue were included in this analysis. Then, case data were collected, including blood routine tests (white blood cell count, eosinophilic percentage, hemoglobin concentration, and platelet count) and erythrocyte sedimentation rate (ESR), computed tomography (CT) or x-ray or magnetic resonance imaging (MRI) examination, and cardiac ultrasound and chest ultrasound. Finally, the following examination data were checked to ensure whether there is any misdiagnosis experience: all children with fever and cough were examined for tuberculous bacteria. Participants with pleural, pericardial and peritoneal effusion underwent puncture. Routine, biochemical, and etiological tests for puncture fluid were performed to exclude other pathogens and tumors.

### 2.3. Statistical methods

SPSS 22.0 was used for statistical analysis. The median was used for continuous variables because of the skewed distribution of data. Proportion (%) was selected for description of categorical variables.

### 2.4. Ethics approval and informed consent

Ethical approval for this study was obtained from the Ethics Committee of Xinhua Hospital affiliated to Shanghai Jiao Tong University School of Medicine (NO. XHEC-D-2022-247). As this was a retrospective study, the requirement of obtaining informed consent was waived.

## 3. Results

### 3.1. Patient demographics and clinical manifestations

A total of 45 children diagnosed with paragonimiasis infection were included in this study. The median age of these children was 96 months (minimum age of 18 months, and maximum age of 16 years). Among these 45 children, 31 children were male (68.9%) and 14 were female (31.1%); 44 children belonged to the Han race (97.8%) and 1 child belonged to the Miao race (2.2%). Majority of these children were from Yanjin county, while 1 child was from a neighboring county (Shuifu).

### 3.2. Clinical manifestations and laboratory features

All of these cases were clinically diagnosed. The clinical classification is shown in Table 1. Among these 45 cases, 25 were of the complex type, 14 were of the thoracopulmonary type, and 6 were of the extrapulmonary type. The clinical symptoms of different clinical types are shown in Supplementary Material S1. Clinical symptoms were closely related to the invaded systems. The most common symptoms were fever, cough, chest pain, and subcutaneous nodules.

**Table 1:** Characteristic of demographic factors and clinical classification.

Variables		*n* (%)
Gender	Male	31 (68.89)
	Female	14 (31.11)
Race	Han	44 (97.78)
	Miao	1 (2.22)
Clinical type	**Thoracopulmonary type**	14 (31.11)
Simple extrapulmonary type		
	Abdominal	1 (2.22)
	Cerebral	1 (2.22)
	Subcutaneous mass	4 (8.89)
Complex type (thoracopulmonary type + extrapulmonary type)		
	Thoracopulmonary + abdominal	3 (6.67)
	Thoracopulmonary + cerebral	3 (6.67)
	Thoracopulmonary + pericardium + subcutaneous mass	8 (17.78)
	Thoracopulmonary + pericardium	3 (6.67)
	Thoracopulmonary + pericardium + abdominal + subcutaneous mass	3 (6.67)
	Thoracopulmonary + pericardium + abdominal	3 (6.67)
	Thoracopulmonary + subcutaneous mass + abdominal	1 (2.22)
Complex type (extrapulmonary type + extrapulmonary type)		
	Subcutaneous mass + pericardium	1 (2.22)

For all children, results of routine blood tests and ESR were performed at the time of their first visit to the hospital (Supplementary Material S2). The median peripheral white blood cell count in venous blood samples was 11.9*10^9^L, and the percentage of eosinophils was elevated in all but one child. ESR was higher than normal in all children. In all children, *Paragonimus* antibody test by the enzyme-linked immunosorbent assay (ELISA) was positive.

**Figure 2:**
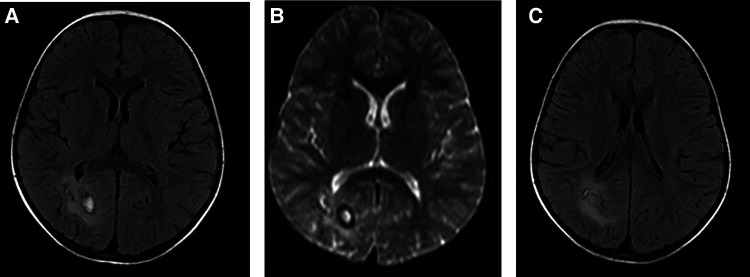
A 5-year-old girl visited our hospital due to limb movement disability. Brain MRI showed the lesion and its surrounding edema (**A, B**), and tunnel-like transitional signs (**C**).

On pulmonary CT, the most common findings were pleural effusion (68.9%) and alveolar exudation (64.4%) (Supplementary Material S3, Figure 1). Brain edema, nodules, and cerebral hemorrhage were found in children with cerebral paragonimiasis (Supplementary Material S3, Figure 2). Pleural effusion was one of the most important findings of ultrasonography. In addition, ultrasound examination also showed abdominal effusion, pericardial effusion, and subcutaneous nodules.

### 3.3. Risk factors

These 45 children had multiple histories of drinking untreated water (*n* = 16, 35.56%), consuming semi-raw/raw freshwater crab meat (*n* = 25, 55.56%), or consuming semi-raw/raw meat (*n* = 10, 22.22%).

### 3.4. The experience of misdiagnosis

Before the diagnosis of paragonimiasis, 6 cases had been misdiagnosed (Table 2). Three patients presented with “fever, cough, and pleural effusion”, and were initially diagnosed as having pulmonary tuberculosis. A 5-year-old girl presented with “walking instability and vomiting”, and she was initially diagnosed with viral encephalitis. Two cases were misdiagnosed as tumors due to “fever, local mass, and multiple serous effusions”. The anti-tuberculosis treatment and anti-infective therapy were not effective, and the diagnosis was confirmed after detailed diet investigation and antibody detection of *Paragonimus*.

**Table 2:** Misdiagnosed patient information list.

No	Age (months)	Clinical presentation	Initial diagnosis	Final diagnosis	Time interval*
1	45	Fever, cough, and chest pain	Tuberculosis	Thoracopulmonary type	14 days
2	32	Fever, cough, and chest pain	Tuberculosis	Thoracopulmonary type	14 days
3	106	Fever, cough, and increased respiratory rate	Tuberculosis	Thoracopulmonary + pericardium	14 days
4	96	Fever and increased respiratory rate	Tumor	Thoracopulmonary + pericardium + abdominal type	10 days
5	98	Fever and increased respiratory rate	Tumor	Thoracopulmonary + pericardium + abdominal type	10 days
6	60	Dysphoria	Viral encephalitis	Cerebral type	28 days

* Time interval between the initial and final diagnosis.

### 3.5. Treatment and follow-up outcomes

Praziquantel (Shenyang Hongqi Pharmaceutical Co. LTD) was the first drug of choice. It was given orally for three days at a dose of 75 mg/kg/day, three times a day, and then it was stopped for four days. The longest course of treatment was 12 weeks in a girl with cerebral paragonimiasis. For children with cerebral paragonimiasis, sedation, mannitol, furosemide and other intracranial pressure-lowering treatments were also given. In addition, adverse reactions to praziquantel, such as anaphylaxis, liver damage, and electrocardiographic abnormalities, were monitored. There was no adverse reaction to praziquantel during treatment.

In 43 cases, the original symptoms were markedly improved and serosal effusion disappeared. Eosinophils and ESR levels returned to normal within three months. The percentage of eosinophils returned to normal. CT or MRI showed narrowing and calcification of the lesion. Most children had a good prognosis, but one child with cerebral paragonimiasis experienced sequelae-limb motor disability. During the follow-up, two children, who were brothers, experienced a re-infection after symptom improvement due to consumption of raw meat again. They were first diagnosed with the thoracopulmonary type; and after treatment, cough symptoms disappeared and chest CT lesions calcified. After about five months, these two patients developed cough and subcutaneous nodules, and the antibody tests of paragonimiasis again turned positive; then, they were diagnosed as having the complex type. These two patients ate raw meat again.

## 4. Discussion

Despite significant improvement in economic and living conditions, paragonimiasis is still one of the diseases that affect the health of children, especially cerebral paragonimiasis. In this study, we retrospectively analyzed the clinical characteristics of 45 children with paragonimiasis in order to improve the recognition of this disease and reduce misdiagnosis.

The clinical manifestations of paragonimiasis in children are diverse and involve many systems, which is closely related to the life history of paragonimiasis. The 45 cases of paragonimiasis included in our study showed 4 clinical subtypes, and many of them were complex and involved multiple systems. The main clinical manifestations included fever, cough, pleural effusion, abdominal effusion, and subcutaneous nodules, which were consistent with previous studies (7). The results of laboratory and imaging examinations were basically consistent with the clinical symptoms.

Being a foodborne parasitic disease, risk factor is very important for establishing the diagnosis of paragonimiasis. The main ways of human infection with *Paragonimus* are drinking untreated water, consuming raw or semi-raw crayfish and freshwater crab, and consuming raw or semi-raw meat. All children included in this survey had a clear habit of drinking untreated water, eating raw crabs or crayfish, or eating uncooked meat, which is consistent with other studies (7–9). It is not easy for children to accurately recall their dietary history, and this can also lead to misdiagnosis or delayed diagnosis. We recommend careful and repeated questioning of the primary caregiver (sometimes not their parents). During follow-up, two boys (brothers) experienced re-infection because they did not change their habit of eating raw meat. Thus, changing inappropriate eating habits is a simple and effective way to prevent paragonimiasis.

Etiological diagnosis is the gold standard for the diagnosis of paragonimiasis. In this study, due to technical limitations, we only conducted ELISA. In this study, all children were positive for the *Paragonimus* antibody. In addition to ELISA detection of antibodies, other methods, such as detection of eggs in sputum or feces, and detection of worms or eggs in subcutaneous nodules, other tissues, or body fluids, are also used for etiological determination.

The diversity and lack of specificity of clinical symptoms, signs, and laboratory tests can easily lead to misdiagnosis. As shown in previous studies, paragonimiasis can be easily misdiagnosed as tuberculosis or cancer (10, 11); 6 cases had been misdiagnosed as tuberculosis, tumor, and viral encephalitis in our study. The radiographic findings, which presented in our results, are also found in other diseases, such as pneumonia, lung neoplasms, tuberculosis, central nervous system neoplasms, vascular diseases, and inflammatory disorders (10, 12, 13). In addition to paragonimiasis, a significant increase in the percentage of eosinophils is also seen in many diseases, such as allergic diseases, other parasitic infections, eosinophilic gastritis, hypereosinophilic syndrome, and mycotic infection (10).

The diagnosis of paragonimiasis requires a combination of symptoms, signs, laboratory tests, risk factor, and other factors. In areas with high incidence of pneumofluke disease, the possibility of paragonimiasis should be considered in patients with multi-system symptoms. It is recommended that patients should be carefully enquired about their dietary habits, and etiological detection of *Paragonimus* should be conducted as soon as possible. If necessary, a tissue biopsy can also be used for establishing the diagnosis (14). CT or MRI may reveal migratory symptoms or the tunnel sign, which are related to the migration of *Paragonimus* larvae, and these findings may be useful to establish the diagnosis (13, 14).

All children were treated with praziquantel and their symptoms improved after treatment. For cerebral paragonimiasis, surgical intervention is necessary if signs of cerebral herniation occur (14). In our study, four children with cerebral paragonimiasis were treated with praziquantel for up to 12 weeks. After treatment, headache, vomiting, and other symptoms were relieved, but a 5-year-old girl experienced sequelae (limb motor disability) due to persistence of cerebral tissue injury. In addition, patients with cerebral paragonimiasis may experience other sequelae, such as speech disorders and seizures (3, 8). Cerebral paragonimiasis is one of the most serious types of paragonimiasis, and it can cause headache, vomiting, movement disorders, seizures, and even death due to intracranial hypertension (3, 15, 16). All four children had symptoms, such as headache and intracranial hypertension, which were consistent with a previous study (14). The main pathogenic mechanism of cerebral paragonimiasis is direct and indirect damage to the brain tissue. *Paragonimu*s larvae migrate to the brain tissue, directly destroying the local brain tissue. They can damage different parts of cerebral tissue, and the clinical symptoms also vary. Indirect injury is mainly caused by local brain edema, followed by intracranial hypertension. At this point, patients may present with symptoms of intracranial hypertension, such as headache, vomiting, papilledema, and hypertension (14).

However, this study has some limitations that should be acknowledged. It was a retrospective study, and a small number of children were included in the study.

## 5. Conclusion

An analysis of the clinical, laboratory, and imaging features of children diagnosed with paragonimiasis is helpful to improve the understanding and diagnosis of paragonimiasis. In children, the clinical manifestations of paragonimiasis are diverse and lack specificity, which can easily lead to misdiagnosis. The characteristic tunnel sign on CT or MRI is helpful for making the diagnosis. Children living in areas with high incidence of paragonimiasis and who have fever, cough, local nodules, and multiple serous effusions should be carefully enquired about their dietary habits. Praziquantel is the first drug of choice for the treatment of paragonimiasis. Most patients recover well after treatment, but severe cases may experience sequelae. People living in areas with high incidence of paragonimiasis should be encouraged to change their dietary patterns to prevent paragonimiasis. Avoiding drinking untreated water and eating cold and raw food is a simple but important measure to prevent paragonimiasis.

## Data Availability

The original contributions presented in the study are included in the article/**Supplementary Material**, further inquiries can be directed to the corresponding author/s.
