# Supplementary material for: Pediatric paragonimiasis: a retrospective analysis of cases from a county in south-west China
Source: Front Pediatr. 2023 May 17;11:1143262. doi: 10.3389/fped.2023.1143262 (PMC10229819; doi:10.3389/fped.2023.1143262)
Supplement: Supplementary file 1 [file Table1.docx]

Supplementary Material

**Pediatric paragonimiasis: a retrospective analysis of cases from a county in south-west China**

**Running title: Retrospective analysis of pediatric paragonimiasis**

Yong-xin Jiang, Gong-qiang Li，Cheng-jing Pan, Zhong-qiu He, Chao Wang, Qi-ru Mu^1^, Lu-lu Cao^*^

^*^ **Correspondence:** Lu-lu Cao

E-mail: caolulu0813@163.com; caolulu@xinhuamed.com.cn

1. **Supplementary Tables**

There are three supplementary tables. Supplementary tables are fllowing:

| **Supp table-1: Clinical manifestations of different clinical types** | | | | | | | | |
| --- | --- | --- | --- | --- | --- | --- | --- | --- |
| Clinical type | **Symptom (n)** | | | | | | | |
|  | fever | headache | cough | chest pain | abdominal pain | epileptic seizure | motor disorders | subcutaneous nodules |
| **Thoracopulmonary type** | 9 | 0 | 10 | 6 | 0 | 0 | 0 | 0 |
| **Extrapulmonary type** |  |  |  |  |  |  |  |  |
| Abdominal type | 0 | 0 | 0 | 0 | 1 | 0 | 0 | 0 |
| Cerebral type | 0 | 1 | 0 | 0 | 0 | 0 | 1 | 0 |
| Subcutaneous mass type | 0 | 0 | 0 | 0 | 0 | 0 | 0 | 4^*^ |
| **Complex type** |  |  |  |  |  |  |  |  |
| Thoracopulmonary type +extrapulmonary type | 6 | 2 | 8 | 2 | 5 | 1 | 0 | 12^**^ |
| Subcutaneous mass + pericardium type | 0 | 0 | 0 | 0 | 0 | 0 | 0 | 1^***^ |
| ALL | 15 | 3 | 18 | 8 | 6 | 1 | 1 | 17 |
|  |  |  |  |  |  |  |  |  |
| * Sites of subcutaneous mass: abdominal wall (n=3), left chest wall (n=1) | | | | | | | | |
| ** Sites of subcutaneous mass: abdominal wall (n=6), neck (n=1), right groin (n=3), right axilla (n=1), and left axilla (n=1) | | | | | | | | |
| *** Sites of subcutaneous mass: right chest wall (n=1) | | | | | | | | |

| **Supp table-2: Results of blood tests (n=45)** | | | | | |
| --- | --- | --- | --- | --- | --- |
|  | **Min** | **Max** | **Percentile** | | |
|  |  |  | ***P25*** | ***P50*** | ***P75*** |
| White blood cell count (10^9/L） | 3.55 | 28.28 | 8.26 | 11.89 | 16.26 |
| Eosinophils（%） | 0.30 | 72.60 | 16.18 | 36.35 | 47.23 |
| Hemoglobin（g/l） | 82.00 | 154.00 | 109.75 | 116.00 | 125.00 |
| Platelet（10^9/L） | 178.00 | 709.00 | 266.00 | 337.00 | 436.50 |
| Erythrocyte sedimentation rate（mm/h） | 8.00 | 111.00 | 25.25 | 45.00 | 67.25 |

| **Supp table-3: Characteristics of imaging findings.** | | |
| --- | --- | --- |
| **Imaging findings** | | **n (%)** |
| CT and MRI | Pulmonary alveoli exudation | 29 (64.44) |
|  | Pleural effusion | 31 (68.89) |
|  | Pericardial effusion | 10 (22.22) |
|  | Pleural thickening | 1 (2.22) |
|  | Pulmonary nodules | 1 (2.22) |
|  | Brain edema | 1 (2.22) |
|  | Cystic lesion | 1 (2.22) |
|  | Cerebral hemorrhage | 2 (4.44) |
|  | Multiple lesions | 2 (2.22) |
|  | Multiple abnormal signals | 2 (2.22) |
| Ultrasound examination | Pleural effusion | 32 (71.11) |
|  | Abdominal cavity effusion | 13 (28.89) |
|  | Abdominal subcutaneous nodules | 6 (13.33) |
|  | Pericardial effusion | 11 (24.44) |
